# Assessing phenotypic effect of integrase strand-transfer inhibitor (INSTI)-based resistance substitutions associated with failures on cabotegravir

**DOI:** 10.1093/jac/dkaf019

**Published:** 2025-01-24

**Authors:** Michelle L D’Antoni, Brie Falkard, Kristen Andreatta, Stephanie Cox, Cal Cohen, Christian Callebaut

**Affiliations:** Clinical Virology, Gilead Sciences, Inc., 333 Lakeside Drive, Foster City, CA 94404, USA; Clinical Virology, Gilead Sciences, Inc., 333 Lakeside Drive, Foster City, CA 94404, USA; Clinical Virology, Gilead Sciences, Inc., 333 Lakeside Drive, Foster City, CA 94404, USA; Clinical Virology, Gilead Sciences, Inc., 333 Lakeside Drive, Foster City, CA 94404, USA; Medical Affairs, Gilead Sciences, Inc., 333 Lakeside Drive, Foster City, CA 94404, USA; Clinical Virology, Gilead Sciences, Inc., 333 Lakeside Drive, Foster City, CA 94404, USA

## Abstract

**Objectives:**

International guidelines recommend integrase strand-transfer inhibitor (INSTI)-based regimens as initial and switch therapy in people with HIV. As novel INSTIs become available, understanding how emergence of resistance at virological failures and seroconversions affects subsequent treatment options is needed. For the latest approved INSTI, cabotegravir, resistance patterns comprising Q148K/R, N155H, R263K, G118R, E138A/K and G140A/S (alone or in combination) have been documented in virological failures and seroconversions. Here, the effect of these substitutions on antiviral activity of commercially approved INSTIs, bictegravir and elvitegravir, was assessed.

**Methods:**

Antiviral testing was performed using person-derived clinical isolates (*n* = 52) with viral profiles similar to cabotegravir INSTI resistance patterns; susceptibility to cabotegravir, bictegravir and elvitegravir was measured using a phenotypic assay. Substitution patterns from isolates included triple [Q148K/H/R + E138A/K + G140A/C/S (*n* = 16)], double [Q148R + E138K (*n* = 3); Q148H/R + G140A/S (*n* = 24)] and single [N155H (*n* = 6); Q148R (*n* = 3)] resistance-associated mutations (RAMs).

**Results:**

IC_50_ fold changes (FCs) for triple RAMs were the highest, at 47.0, 7.59 and >144 for cabotegravir, bictegravir and elvitegravir, respectively. For cabotegravir, bictegravir and elvitegravir, respectively, mean IC_50_ FCs were 9.5, 2.5 and >144 for double RAMs; and 3.3, 1.4 and >65 for single RAMs. When considering clinical/biological assay cut-offs, 54% (28/52) of isolates were susceptible to bictegravir, 40% (21/52) were partially susceptible and 6% (3/52) were resistant; for elvitegravir, 100% of isolates were resistant. Cabotegravir cut-offs were not available at the time of reporting.

**Conclusions:**

Overall, clinical isolates with RAM patterns similar to clinically observed cabotegravir INSTI resistance showed meaningful increases in IC_50_ FCs, suggesting that cabotegravir-associated resistance may negatively affect efficacy of bictegravir- and elvitegravir-based regimens.

## Introduction

HIV-1 treatment guidelines recommend integrase strand-transfer inhibitor (INSTI)-based regimens for both initial and switch ART in people with HIV (PWH).^1–3^ As novels agents are introduced for HIV-1 treatment and pre-exposure prophylaxis (PrEP), it is important to understand resistance emergence at virological failures and seroconversions, as cross-resistance can limit subsequent treatment options. Guidelines also recommend that initial regimen selection should be individualized based on factors including resistance test results and prior exposure to particular PrEP agents.^1^

Long-acting injectable cabotegravir, partnered with long-acting injectable rilpivirine, is now approved in multiple countries for treatment of virologically suppressed PWH. Cabotegravir/rilpivirine has been studied in Phase 3 clinical trials of PWH with >6 months of virological suppression with dosing intervals of 4 and 8 weeks [4 weeks (Q4W): ATLAS^4^; 8 weeks (Q8W: ATLAS-2M^5,6^ and SOLAR^7^) and in participants who initiated oral ART for 20 weeks and then received Q4W injections (FLAIR^8^). Additionally, cabotegravir/rilpivirine has also been investigated in Phase 3 randomized open-label trials, which included participants with a history of adherence challenges [Q4W: LATITUDE (ACTG A5359)]^9^ and virologically suppressed participants from Uganda, Kenya and South Africa (Q8W: CARES).^10^ Single-agent cabotegravir is also approved for PrEP in several countries, and was supported by clinical trials in cisgender men and transgender women (HPTN 083^11^) and cisgender women (HPTN 084^12^).

Overall in clinical trials, few events of virological failure (∼1%) after cabotegravir/rilpivirine treatment (48–152 weeks of follow-up) or seroconversions (∼0.5%) with cabotegravir PrEP usage have been reported. However, INSTI resistance has been documented at relatively high levels, including the primary integrase resistance-associated mutations (RAMs) Q148R, N155H, R263K and G118R, alone or in combination with secondary integrase RAMs in participants who experienced virological failure after switch, or in participants who were diagnosed with HIV-1 infection after PrEP administration.^13^ Currently, there remains uncertainty regarding the impact of these newly reported INSTI RAM patterns associated with cabotegravir virological failures/seroconversions on other drugs of the same class. Here we examined the potential effect of INSTI RAMs linked to cabotegravir virological failures/seroconversions on antiviral activity of the commercially approved INSTIs bictegravir and elvitegravir.

## Materials and methods

### Clinical isolates

**Figure 1.**
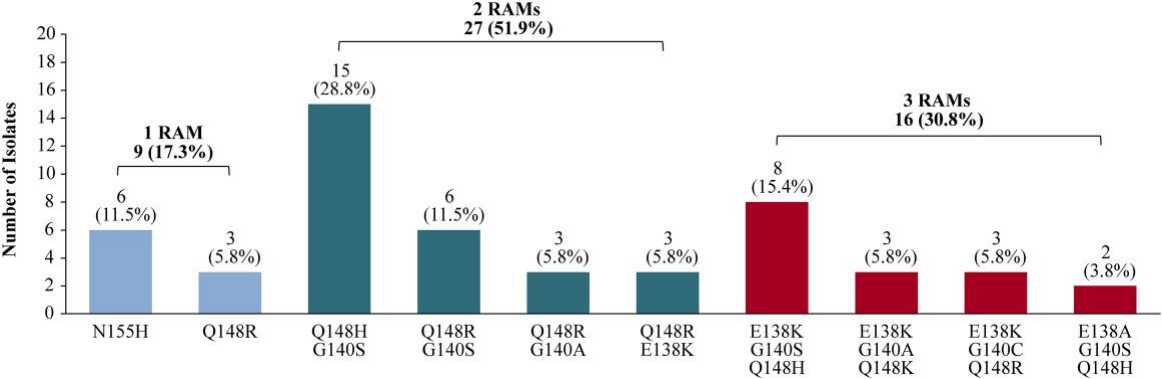
INSTI RAM patterns from selected clinical isolates (*n* = 52).

Clinical isolates derived from plasma samples from treatment-experienced PWH were selected from a biobank collection from Monogram Biosciences (South San Francisco, CA, USA). Viral profiles, which were determined using the GeneSeq HIV Integrase assay (Monogram Biosciences), included the same primary INSTI RAMs detected in cabotegravir failures or seroconversions (Figure 1; Table S1, available as Supplementary data at *JAC* Online).

### Antiviral testing

Cabotegravir, bictegravir and elvitegravir were provided by Monogram Biosciences. Phenotype of clinical isolates was determined using the PhenoSense^®^ Integrase assay (Monogram Biosciences) and their susceptibility to the INSTIs was assessed. IC_50_ was determined for each clinical isolate and expressed as a fold change (FC) relative to WT virus.

## Results

### INSTI resistance substitution patterns

Of the 52 clinical isolates available for this study, over half had two INSTI RAMs and almost a third had three INSTI RAMs. Patterns included single mutants with primary N155H (*n* = 6) and Q148R (*n* = 3) substitutions, double mutants with combinations including the primary Q148 substitution with a secondary mutation, specifically Q148R + E138K (*n* = 3) and Q148H/R + G140A/S (*n* = 24) and triple mutants with the primary Q148 substitution and two secondary mutations: Q148K/H/R + E138A/K + G140A/C/S (*n* = 16; Figure 1; Table S1).

### Antiviral activity

**Figure 2.**
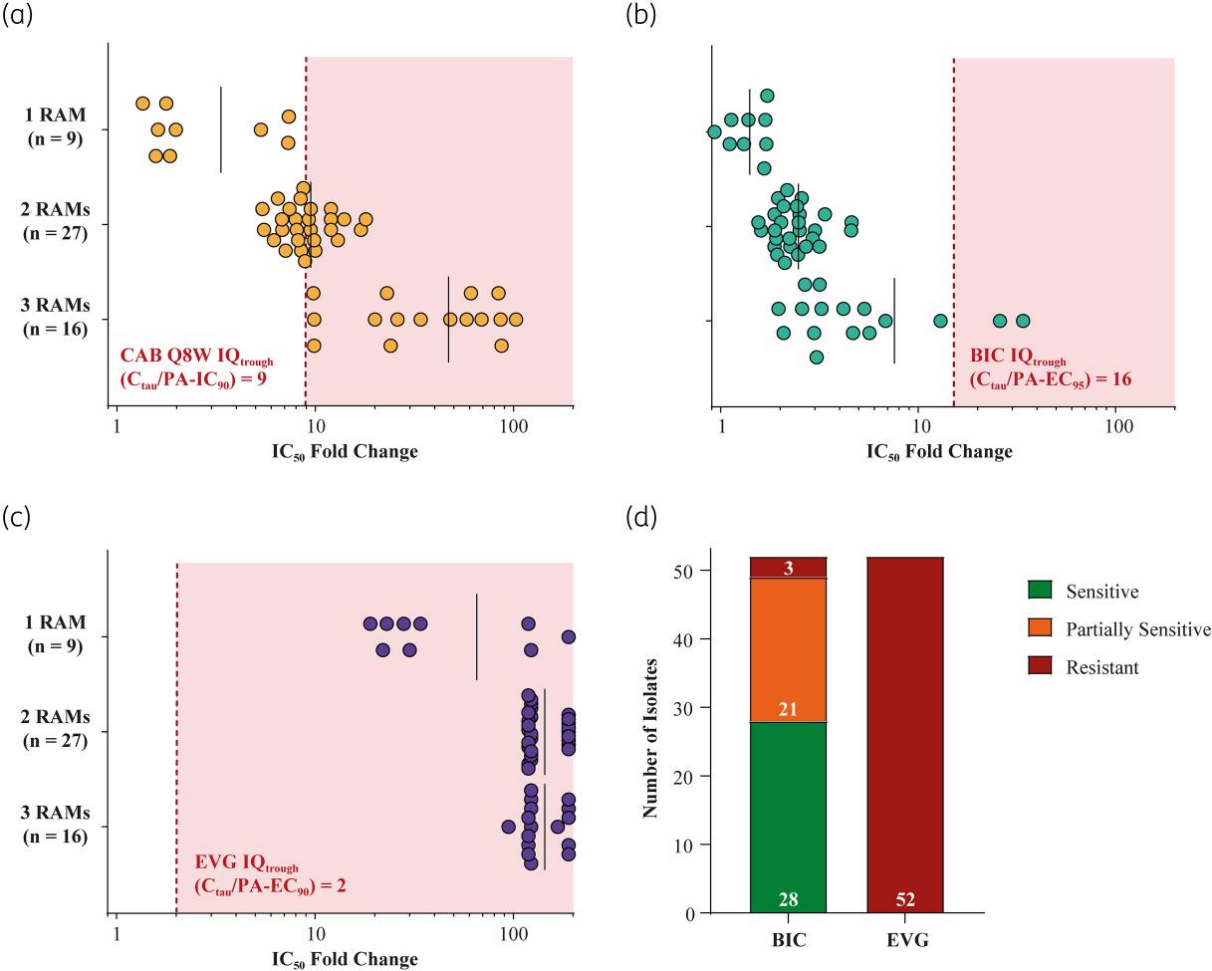
IC50 FC for clinical isolates with RAM patterns associated with cabotegravir failures—(a) cabotegravir (CAB), (b) bictegravir (BIC), (c) elvitegravir (EVG); and (d) assessment of clinical isolate susceptibility to BIC and EVG. In panels (a) to (c), vertical black bars represent mean FCs; red dashed lines indicate IQ values. Substitution patterns from isolates: Q148K/H/R + E138A/K + G140A/C/S (n = 16); Q148R + E138K (n = 3); Q148H/R + G140A/S (n = 24); N155H (n = 6); Q148R (n = 3). In panel (d), for BIC, FCs from 2.5 to 10 signified partial susceptibility and fold changes >10 signified resistance; for EVG, FCs of >2.5 indicated resistance.

Phenotypic susceptibility of clinical isolates was determined using IC_50_ FCs, which were grouped based on the number of INSTI RAMs present in the clinical isolates, for each of the three INSTIs (Figure 2a–c). Mean IC_50_ FCs for triple RAMs were the highest, at 47.0, 7.59 and >144 for cabotegravir, bictegravir and elvitegravir, respectively (Figure 2a–c). For cabotegravir, bictegravir and elvitegravir, respectively, mean IC_50_ FCs were 9.5, 2.5 and >144 for double RAMs, and 3.3, 1.4 and >65 for single RAMs (Figure 2a–c). IC_50_ FCs for individual clinical isolates are found in Table S2.

Measured IC_50_ FCs were compared with published clinical trough concentrations [expressed as inhibitory quotient (IQ_trough_)], to explore whether *in vivo* drug levels could potentially inhibit viral replication for clinical context. For cabotegravir, the IQ_trough_ for the Q8W long-acting injectable cabotegravir/rilpivirine regimen was used, which is 9 [trough concentration (*C*_tau_)/protein-adjusted (PA) IC_90_].^14^ For bictegravir, the IQ_trough_ for the single-tablet regimen (STR) bictegravir/emtricitabine/tenofovir alafenamide (BIC/FTC/TAF) was used, which is 16 (*C*_tau_/PA EC_95_).^15,16^ For elvitegravir, the IQ_trough_ for the STR elvitegravir/cobicistat/emtricitabine/tenofovir disoproxil fumarate was used, which is 2 (*C*_tau_/PA EC_95_).^16,17^ Overall, of the 52 isolates tested, 58% of cabotegravir IC_50_ FCs had a higher numerical value than the IQ_trough_ of 9. In comparison, only 4% of the bictegravir IC_50_ FCs were greater than the IQ_trough_ of 16. For IC_50_ FCs for elvitegravir, 100% were numerically higher than the elvitegravir IQ_trough_ of 2 (Figure 2a–c).

### Assay sensitivity assessment

When considering clinical and biological assay cut-offs reported by the phenotyping assay, 54% (28/52) of isolates were categorized as susceptible to bictegravir, 40% (21/52) were partially susceptible (i.e. partial antiviral activity) and 6% (3/52) were resistant (Figure 2d). For elvitegravir, 100% of isolates were reported as resistant (Figure 2d). Published cut-offs for cabotegravir were not available at the time the data were generated.

## Discussion

Although currently INSTI resistance is rarely observed in HIV treatment and prevention, studying integrase resistance remains important and relevant as INSTIs are the backbone of initial and switch regimens for most PWH. Furthermore, recent reports suggest that INSTI resistance rates are increasing and have the potential to increase even further, which underscores the need to characterize INSTI cross-resistance. In surveillance studies conducted in low- and middle-income countries (such as Malawi, Mozambique, Uganda and Ukraine), prevalence of dolutegravir resistance among individuals with detectable viraemia receiving dolutegravir-based ART has surpassed what has been observed in clinical trials (3.9%–19.6%).^18^ The DTG RESIST study^19^ utilizing data from Southern Africa, Europe and North America also reported high levels of dolutegravir resistance in 6.0% of participants, with increased risk in individuals receiving monotherapy or dolutegravir plus lamivudine dual therapy.^19^ Additionally, an HIV epidemic model investigating the effects of cabotegravir PrEP introduction in sub-Saharan Africa showed that INSTI resistance after 20 years could be 13.1% with the cabotegravir PrEP introduction compared with 1.7% without.^20^

The observation of treatment-emergent INSTI resistance in clinical trials with the newest INSTI, cabotegravir, is notably higher than with bictegravir and dolutegravir, and there is consequently a need to understand the clinical implications of subsequent ART options. In this analysis, clinical isolates with RAM patterns similar to observed INSTI resistance linked to cabotegravir virological failures/seroconversions showed meaningful increases in IC_50_ FCs, which strongly reduced susceptibility to elvitegravir and, to a lesser extent, bictegravir. Comparing the increased IC_50_ FCs with clinical trough concentrations can aid in understanding the impact of the RAM patterns in PWH. For a proportion of the isolates, *in vivo* bictegravir drug levels may not offer appropriate antiviral activity and this observation is even more pronounced for elvitegravir, which would not provide adequate suppression of viral replication in these variants. These observations are in line with the barrier to resistance of earlier INSTIs like elvitegravir and raltegravir, which are lower compared with subsequent INSTIs, like bictegravir, dolutegravir and cabotegravir. The impact of RAMs emerging from cabotegravir on dolutegravir have been previously examined. In a systemic review of cabotegravir PrEP data, RAMs were re-analysed using the Stanford HIV Drug Resistance Database (V9.2) and of the seven INSTI patterns identified, six had predicted intermediate dolutegravir resistance and one high dolutegravir resistance.^21^ It is important to note that guidelines do not recommend the use of bictegravir after failure on an INSTI-containing regimen as the effective dose of bictegravir in these situations is unknown.^1^ However, in the presence of certain INSTI mutations, dolutegravir can be used twice daily (TIVICAY).^1,22^

The fact that virological failure samples from cabotegravir-treated individuals were unavailable for testing is a limitation of this study. Instead, patient-derived viruses with similar primary RAM patterns previously described were analysed. These isolates had unique integrase genetic backgrounds (i.e. different secondary mutations, accessory and polymorphic substitutions), which may have affected viral fitness and potentially drug susceptibility. However, in ATLAS-2M, where comparisons of phenotypic data were possible, half of the IC_50_ FC values were higher for cabotegravir than bictegravir,^5^ supporting the validity of this study. Additionally, our selected isolates for this analysis did not cover all RAMs observed in cabotegravir clinical trials (Table S1). Another unaddressed point is the impact of undocumented minority RAM variants, which could be present and emerge at detectable frequencies under drug pressure, potentially further affecting drug susceptibility; currently, data are lacking on the clinical relevance of RAMs reported using deep-sequencing levels below 15% frequency (population sequencing levels). Moreover, the phenotyping assay sensitivity assessment for cabotegravir was not available at the time of testing but mean IC_50_ FC values for double and triple mutants of 9.5 and 47, respectively, indicate that there was significant resistance associated with the INSTI RAMs. Even the single mutation Q148R had IC_50_ FC values between 5 and 7, suggesting a high level of resistance. Lastly, real-world outcomes of switches to INSTI-based regimens after failure on a cabotegravir regimen are lacking.

For PWH who have a history of cabotegravir use as PrEP, guidelines recommend utilization of a regimen using boosted darunavir with two NRTIs, pending INSTI genotypic resistance testing results.^1^ Experience to guide therapy upon failure of INSTI plus non-NRTI regimens is limited but treatment strategies should be based on past treatment history, drug resistance test results and the potential potency of the next regimen.^1^ In the presence of INSTI resistance, fully active commonly used antiretroviral drug options can include a boosted protease inhibitor with two NRTIs.^1,2^ While boosted PIs have a high barrier to resistance, regimens with ritonavir or cobicistat as CYP3A inhibitors are at higher risk of drug–drug interactions, adverse events and laboratory abnormalities, making clinical management potentially more challenging. Future regimen selection can be additionally limited in individuals receiving cabotegravir/rilpivirine as approximately half of the clinical trial participants experiencing virological failure had dual-class resistance.^4–10^ Moreover, treatment-emergent NNRTI resistance alone may negatively impact subsequent treatment options, including INSTI-based regimens. While the mechanism driving this observation is not defined, data from the ADVANCE study in South Africa demonstrated that virological failure on a dolutegravir-based regimen was potentially impacted by baseline NNRTI resistance.^23^

In conclusion, these data suggest that resistance associated with cabotegravir virological failures/seroconversions could likely negatively affect the efficacy of elvitegravir-based regimens, including elvitegravir/cobicistat/emtricitabine/tenofovir disoproxil fumarate and elvitegravir/cobicistat/emtricitabine/tenofovir alafenamide, and may negatively affect the efficacy of bictegravir-based regimens, specifically BIC/FTC/TAF. These data reinforce recent guideline updates to test for INSTI resistance after treatment or PrEP failure under cabotegravir.^1^ These data also highlight the need for careful selection of subsequent treatment regimens in people with cabotegravir resistance, as other INSTIs may not be fully effective.

## Supplementary Material

dkaf019_Supplementary_Data

## Supplementary data

Tables S1 and S2 are available as Supplementary data at *JAC* Online.
