# Supplementary material for: Assessing phenotypic effect of integrase strand-transfer inhibitor (INSTI)-based resistance substitutions associated with failures on cabotegravir
Source: J Antimicrob Chemother. 2025 Jan 24;80(4):962–6. doi: 10.1093/jac/dkaf019 (PMC11962372; doi:10.1093/jac/dkaf019)
Supplement: dkaf019_Supplementary_Data [file dkaf019_supplementary_data.docx]

**Supplemental Data**

Supplemental Table 1: Integrase strand transfer inhibitor (INSTI) resistance-associated mutation (RAM) patterns

| RAMs from analysis | n | Documented INSTI resistance in cabotegravir clinical trials | | |
| --- | --- | --- | --- | --- |
|  |  | Mutations pattern | Specific RAMs | Study |
| N155H | 6 | N155H -/+ other mutation | N155H; N155H + S230R; N155H + T97A | HPTN 083; ATLAS; ATLAS-2M |
| Q148R | 3 | Q148R -/+ other mutation | Q148R; Q148R + N155H; Q148R + L74I | HPTN 083; FLAIR; ATLAS-2M; SOLAR |
| E138K, Q148R | 3 | Q148K/R + E138A/K -/+ G140A/S -/+ other mutations | Q148R + E138A/K; Q148K + E138K+ M50I; Q148R + E138K + G140A + M50I; Q148R + E138K + G140S + E157Q + L74I + Q146R | HPTN 083; ATLAS-2M |
| G140A, Q148R | 3 |  |  |  |
| G140S, Q148H | 15 |  |  |  |
| G140S, Q148R | 6 |  |  |  |
| E138K, G140A, Q148K | 3 |  |  |  |
| E138K, G140C, Q148R | 3 |  |  |  |
| E138K, G140S, Q148H | 8 |  |  |  |
| G140S, Q148H, E138A | 2 |  |  |  |

Other mutations patterns from HPTN 083, FLAIR and SOLAR: Q148R + N155H; Q148R + L74I; Q148R + N155H + R263K + Q146L; R263K; N155H + R263K; R263K+ M50I; G140R; V151I; T97A; L74I; M50I; G118R; Q148R + N155H + R263K + Q146L

INSTI, integrase strand transfer inhibitor; RAM, resistance-associated mutation

Supplemental Table 2: Raw IC_50_-FCs for cabotegravir (CAB), bictegravir (BIC), elvitegravir (EVG)

| 1 RAM (n=9) | IC_50_ fold change | | | | | 2 RAMs (n=27) | IC_50_ fold change | | | 3 RAMs (n=16) | IC_50_ fold change | | | | |
| --- | --- | --- | --- | --- | --- | --- | --- | --- | --- | --- | --- | --- | --- | --- | --- |
|  | CAB | | BIC | | EVG |  | CAB | BIC | EVG |  | CAB | | BIC | | EVG |
| N155H | 1.57 | | 1.11 | | 28 | E138K, Q148R | 7.96 | 1.60 | > 123 | E138K, G140A, Q148K | 61 | | 13 | | > 123 |
| N155H | 1.35 | | 0.93 | | 22 | E138K, Q148R | 10 | 1.95 | > 190 | E138K, G140A, Q148K | 103 | | 26 | | > 190 |
| N155H | 1.61 | | 1.7 | | 30 | E138K, Q148R | 8.07 | 1.87 | > 119 | E138K, G140A, Q148K | 84 | | 34 | | > 119 |
| N155H | 1.77 | | 1.31 | | 23 | G140A, Q148R | 5.51 | 1.55 | > 123 | E138K, G140C, Q148R | 58 | | 4.18 | | > 123 |
| N155H | 1.98 | | 1.68 | | 34 | G140A, Q148R | 6.19 | 2.25 | > 190 | E138K, G140C, Q148R | 86 | | 5.67 | | > 190 |
| N155H | 1.85 | | 1.13 | | 19 | G140A, Q148R | 6.48 | 1.87 | > 119 | E138K, G140C, Q148R | 87 | | 4.68 | | > 119 |
| Q148R | 5.32 | | 1.38 | | > 123 | G140S, Q148H | 5.41 | 1.93 | > 123 | E138K, G140S, Q148H | 23 | | 2.08 | | > 123 |
| Q148R | 7.35 | | 1.72 | | > 190 | G140S, Q148H | 8.49 | 2.02 | > 123 | E138K, G140S, Q148H | 9.86 | | 1.96 | | > 123 |
| Q148R | 7.3 | | 1.66 | | > 119 | G140S, Q148H | 7.41 | 1.91 | > 123 | E138K, G140S, Q148H | 20 | | 3.06 | | > 95 |
| **Mean** | **3.3** | | **1.4** | | **> 65.3** | G140S, Q148H | 8.71 | 1.88 | > 123 | E138K, G140S, Q148H | 24 | | 2.67 | | > 190 |
|  |  |  | |  | | G140S, Q148H | 12 | 2.11 | > 123 | E138K, G140S, Q148H | 9.78 | | 2.97 | | > 190 |
|  |  |  | |  | | G140S, Q148H | 6.81 | 2.23 | > 190 | E138K, G140S, Q148H | 34 | | 3.24 | | > 167 |
|  |  |  | |  | | G140S, Q148H | 12 | 2.51 | > 190 | E138K, G140S, Q148H | 26 | | 3.17 | | > 119 |
|  |  |  | |  | | G140S, Q148H | 9.47 | 3 | > 190 | E138K, G140S, Q148H | 9.87 | | 2.58 | | > 119 |
|  |  |  | |  | | G140S, Q148H | 8.19 | 2.51 | > 190 | G140S, Q148H, E138A | 48 | | 5.34 | | > 123 |
|  |  |  | |  | | G140S, Q148H | 13 | 2.46 | > 190 | G140S, Q148H, E138A | 69 | | 6.84 | | > 190 |
|  |  |  | |  | | G140S, Q148H | 6.79 | 2.17 | > 119 | **Mean** | **47** | | **7.59** | | **> 144** |
|  |  |  | |  | | G140S, Q148H | 9.27 | 2.48 | > 119 |  |  |  | |  | |
|  |  |  | |  | | G140S, Q148H | 8.44 | 2.92 | > 119 |  |  |  | |  | |
|  |  |  | |  | | G140S, Q148H | 9.86 | 2.7 | > 119 |  |  |  | |  | |
|  |  |  | |  | | G140S, Q148H | 14 | 2.57 | > 119 |  |  |  | |  | |
|  |  |  | |  | | G140S, Q148R | 12 | 2.43 | > 123 |  |  |  | |  | |
|  |  |  | |  | | G140S, Q148R | 7.09 | 2.08 | > 123 |  |  |  | |  | |
|  |  |  | |  | | G140S, Q148R | 17 | 4.6 | > 190 |  |  |  | |  | |
|  |  |  | |  | | G140S, Q148R | 8.88 | 3.37 | > 190 |  |  |  | |  | |
|  |  |  | |  | | G140S, Q148R | 18 | 4.58 | > 119 |  |  |  | |  | |
|  |  |  | |  | | G140S, Q148R | 9.48 | 3.16 | > 119 |  |  |  | |  | |
|  |  |  | |  | | **Mean** | **9.5** | **2.5** | **> 144** |  |  |  | |  | |

Phenotypic assessment using clinical and biological assay cutoffs is reported as sensitive (green cells), partially sensitive (orange cells) or resistant (red cells). White cells delineate no phenotypic assessment available.

BIC, bictegravir; CAB, cabotegravir; EVG, elvitegravir; fold-change, FC; IC_50_, half-maximal inhibitory concentration, RAM, resistance associated mutation
